# Septal Myectomy and Subvalvular Repair in Hypertrophic Cardiomyopathy, a Systematic Review and Pooled Analysis

**DOI:** 10.31083/j.rcm2409268

**Published:** 2023-09-22

**Authors:** Ming-Yang Song, Xiang Wei, Chen-He Li, Rui Li

**Affiliations:** ^1^Division of Cardiothoracic and Vascular Surgery, Tongji Hospital, Tongji Medical College, Huazhong University of Science and Technology, 430030 Wuhan, Hubei, China; ^2^Key Laboratory of Organ Transplantation, Ministry of Education, 430010 Wuhan, Hubei, China; ^3^NHC Key Laboratory of Organ Transplantation, 430073 Wuhan, Hubei, China; ^4^Key Laboratory of Organ Transplantation, Chinese Academy of Medical Sciences, 430010 Wuhan, Hubei, China

**Keywords:** hypertrophic cardiomyopathy, mitral valve insufficiency, subvalvuar repair, septal myectomy

## Abstract

**Background:**

Some patients with hypertrophic obstructive cardiomyopathy (HOCM) still exhibit systolic anterior motion (SAM) and mitral regurgitation (MR) even after undergoing an isolated ventricular septectomy. Currently, there are disputes regarding whether to perform a mitral valve intervention and which type of operation is more effective.

**Methods:**

By searching PubMed, Cochrane, Embase, Web of Science, FDA.gov, and ClinicalTrials.gov, as well as other resource databases, we obtained all articles published before December 2022 on ventricular septal myectomy combined with mitral valve intervention for hypertrophic cardiomyopathy. Demographic information and outcome variable data were extracted from 10 screened studies on ventricular septal resection combined with mitral valve repair. The risk of bias was assessed using methodological index for non-randomized studies (MINORS). Student’s *t*-test was used for comparisons of continuous variables, and the chi-square or Fisher’s exact test was used for dichotomous variables. A total of 692 patients across 10 studies were analyzed.

**Results:**

There were 5 (0.7%) deaths in the perioperative period. The average cardiopulmonary bypass time was 64.7 ± 22.2 minutes, and the average follow-up time was 39.6 ± 36.3 months. Compared with baseline levels, the left ventricular outflow tract gradient (83.6 ± 32.2 mmHg vs. 11.0 ± 7.8 mmHg, *p* < 0.01), maximum interventricular septal thickness (22.5 ± 5.1 mm vs. 14.7 ± 5.5 mm, *p* < 0.01), III/IV mitral regurgitation (351/692 vs. 17/675, *p* < 0.01), anterior mitral leaflet (AML)-annulus ratio (0.49 ± 0.14 vs. 0.60 ± 0.12, *p* < 0.01), tenting area (2.72 ± 0.60 ${\mathrm{cm}}^{2}$ vs. 1.95 ± 0.60 ${\mathrm{cm}}^{2}$, *p* < 0.01), and SAM (181/194 vs. 11/215,* p* < 0.01) were significantly improved. 14 (2.1%) patients were in New York Heart Association functional class III/IV, which was significantly improved compared with the preoperative state (541/692 vs. 14/682, *p* < 0.01).

**Conclusions:**

Ventricular septectomy combined with mitral valve repair can be a safe and effective treatment option for patients suffering from HOCM with SAM and severe MR.

## 1. Introduction

Hypertrophic obstructive cardiomyopathy (HOCM) is a hereditary disease characterized by left ventricular outflow tract obstruction, the systolic anterior motion of the mitral valve, and moderate to severe mitral regurgitation, with typical clinical symptoms such as dyspnea, angina pectoris, and syncope. The incidence of HOCM is about 0.2% [1], and the disease is associated with a high risk of sudden death. Ventricular septal resection is currently the most common and effective treatment for HOCM in patients whose clinical symptoms cannot be improved by drugs [2, 3]. It has a good effect on relieving left ventricular outflow tract obstruction, relieving symptoms, improving quality of life, and reducing the risk of sudden death.

The main cause of HOCM is abnormal hypertrophy of the ventricular septum, and abnormalities of the anterior mitral valve leaflet, papillary muscle, and secondary chordae may also play an important role in its pathogenesis [4, 5]. These abnormal structures may bind the anterior leaflet of the mitral valve, making it difficult to completely improve the outflow tract obstruction and mitral regurgitation by isolated septal myectomy. About 2.5% of HOCM patients have residual left ventricular outflow tract gradient after septal myectomy [6]. For patients with severe left ventricular outflow tract obstruction accompanied by obvious systolic anterior motion of the mitral valve and mitral valve regurgitation, there may be a mitral valve, papillary muscle, or chordal abnormalities that are difficult to accurately assess by preoperative echocardiography, and septal resection combined with mitral valve surgery may be considered [7]. However, whether combined mitral valve surgery is necessary and what the best mitral valve surgery method is still controversial. The mainstream mitral valve repair includes plication or extension of the anterior leaflet [8, 9], secondary chordal cutting [10], papillary muscle reorientation [11], and edge-to-edge repair [10].

In this study, we integrated patient characteristics, preoperative, postoperative, and follow-up clinical and echocardiographic findings from published reports on septal myectomy combined with sub-mitral valve repair for HOCM. Our aim is to determine whether septal resection combined with subvalvular management can improve clinical outcomes and reduce the incidence of adverse events in patients with HOCM.

## 2. Materials and Methods

### 2.1 Search Strategy and Selection Criteria

This systematic review is reported following the Preferred Reporting Items for Systematic Reviews and Meta-Analyses [12, 13] and was registered on the International Platform of Registered Systematic Review and Meta-Analysis Protocols (number INPLASY202320116). We selected relevant studies published before December 2022 by searching PubMed, Cochrane, Embase, Web of Science, FDA.gov, and ClinicalTrials.gov, with no language restrictions. We used the following combined text and MeSH terms: (((((((((((((((Insufficiency, Mitral Valve) OR (Valve Insufficiency, Mitral)) OR (Mitral Valve Regurgitation)) OR (Regurgitation, Mitral Valve)) OR (Valve Regurgitation, Mitral)) OR (Mitral Regurgitation)) OR (Regurgitation, Mitral)) OR (Mitral Valve Incompetence)) OR (Incompetence, Mitral Valve)) OR (Valve Incompetence, Mitral)) OR (Mitral Incompetence)) OR (Incompetence, Mitral)) OR (Mitral Insufficiency)) OR (Insufficiency, Mitral)) OR (“Mitral Valve Insufficiency”[Mesh])) AND ((“Cardiomyopathy, Hypertrophic”[Mesh]) OR (((((((((Cardiomyopathies, Hypertrophic) OR (Hypertrophic Cardiomyopathies)) OR (Hypertrophic Cardiomyopathy)) OR (Cardiomyopathy, Hypertrophic Obstructive)) OR (Cardiomyopathies, Hypertrophic Obstructive)) OR (Hypertrophic Obstructive Cardiomyopathies)) OR (Hypertrophic Obstructive Cardiomyopathy)) OR (Obstructive Cardiomyopathies, Hypertrophic)) OR (Obstructive Cardiomyopathy, Hypertrophic))).

### 2.2 Data Abstraction and Quality Assessment

**Fig. 1.**
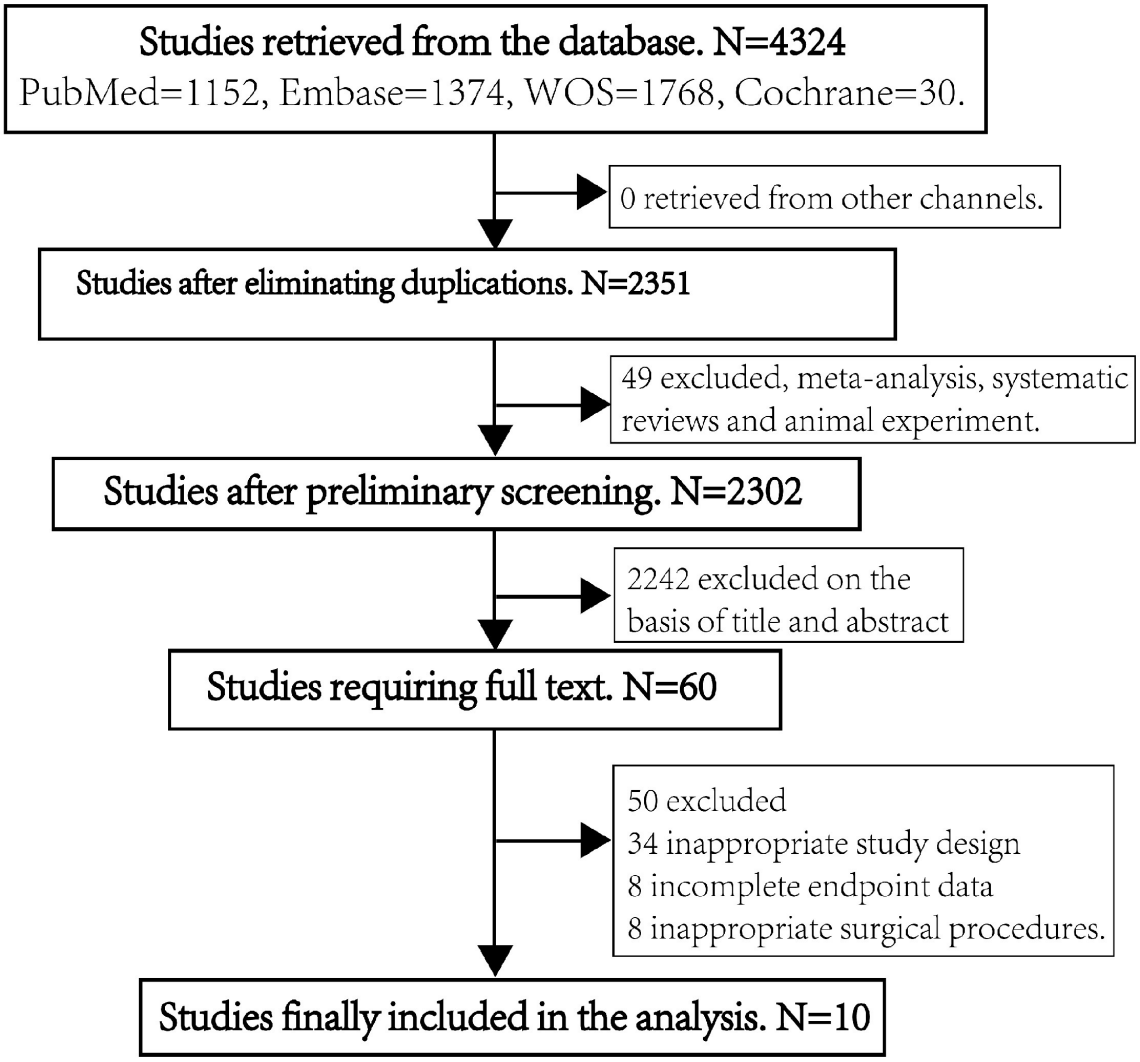
**Study selection process**.

Two independent researchers screened relevant literature by reviewing titles and abstracts. Any disagreements that arose were resolved by a third researcher through negotiation. After the initial screening, the full literature was obtained and further examined. The inclusion criteria for the study were: (1) patients with clinically symptomatic HOCM; and (2) myectomy combined with sub-mitral valve repair was performed. Exclusion criteria included clinical studies of isolated septal myectomy, incomplete clinical and echocardiographic data, outcome variables that did not fit the study’s purpose, and study types such as reviews, case reports, and animal experiments. When multiple studies were published by the same author in different years, only the latest one was included (see Fig. 1). We used the MINORS tool to assess the risk of bias and the quality of the included studies (**Supplementary Table 1**) [14].

Two researchers independently reviewed and screened to extract relevant study population baseline data, including patient number, age, sex, medication history, New York Heart Association functional class, implantable cardioverter defibrillator (ICD) implantation, and related surgical history. They also noted any abnormal papillary muscle and chordae found during operation and treatment techniques, preoperative and postoperative echocardiographic indicators, such as left ventricular diastolic diameter, ejection fraction, interventricular septal thickness, maximum left ventricular outflow tract pressure gradient, mitral valve regurgitation degree, and pacemaker implantation. Additionally, they recorded perioperative mortality, reoperation rates during the follow-up period, and New York Heart functional class.

### 2.3 Statistical Analysis

A systematic review was performed using Review Manager 5.4 (Nordic Cochrane Center, Copenhagen, Denmark). Continuous variables were reported as mean ±SD, while categorical variables were reported as frequencies (percentages). The Student *t*-test was used to compare continuous variables, and the chi-square or Fisher exact test was used for dichotomous variables. All statistical tests were two-sided, with a significance level set at 0.05. The results were analyzed using SPSS 25 software (IBM Corp., Armonk, NY, USA).

## 3. Results

### 3.1 Patient Characteristics

The study included 692 patients, of whom 52.2% were male. The average age was 53.0 ± 14.8. Almost all patients (95.3%) had received the optimal dose of β-blockers or ${\mathrm{Ca}}^{2+}$-blockers before surgery, but their symptoms did not significantly improve. Hypertension was present in 39% of patients (as reported in two studies [15, 16]), and atrial fibrillation was detected on the electrocardiography (ECG) in 25.2% of patients. Before the operation, 78.2% of patients were in New York Heart Association cardiac function class III/IV. ICD implantation was performed in 24 out of 122 patients (19.7%) (reported in two studies [17, 18]). A family history of hypertrophic cardiomyopathy was reported in 27.2% of patients (reported in three studies [15, 17, 18]). All patients met the surgical indications recommended by the guidelines [2, 3]: Left ventricular outflow tract gradient ≥50 mmHg at rest or during provocation; unresponsive to treatment with beta-blockers or ${\mathrm{Ca}}^{2+}$-blockers; echocardiographically measured posterior interventricular septum (IVS) ≥15 mm. Three studies excluded patients with organic mitral valve disease (rheumatic, degenerative, annular calcification, direct insertion of papillary muscle into the mitral valve, papillary muscle displacement), concomitant other valvular disease requiring intervention, history of alcohol septal ablation, and secondary hypertrophic cardiomyopathy due to aortic stenosis or hypertension [10, 15, 19]. Subvalvular structural abnormalities in patients were statistically assessed in a study (n = 56): anomalous papillary muscles (n = 45 or 80.3%), direct insertion into the anterior mitral leaflet (n = 13 or 23.2%), fusion to the ventricular septum (n = 31 or 55.4%), fusion to the left ventricular free wall (n = 12 or 21.4%), accessory papillary muscle (n = 2 or 3.6%), anomalous chordae tendineae (false cords) (n = 28 or 50.0%), the fusion of the mitral leaflet to the septum (n = 3 or 5.4%) [17]. Additionally, the mitral valve and subvalvular apparatus abnormalities are mostly diagnosed directly during operation (n = 30 or 66.7%), but the success rate of diagnosis by Doppler echocardiography is low (n = 15 or 33.3%), indicating that even for professional ultrasound, preoperative diagnosis of mitral valve apparatus abnormalities is also very difficult for cardiologists [20] (Table 1, Ref. [10, 15, 16, 17, 18, 19, 20, 21, 22, 23]).

**Table 1.** **Demographics and clinical characteristics of patients**.

Author	N	Age	Male	Hypertension	Atrial fibrillation	NYHA class III/IV	${\mathrm{Beta/Ca}}^{2+}$ blockers	Family history
Afanasyev 2021 [10]	24	54.1 ± 12.3	14 (58.3%)	NA	NA	24 (100%)	24 (100%)	NA
Liu 2022 [15]	40	53.7 ± 11.4	15 (37.5%)	15 (37.5%)	5 (12.5%)	37 (92.5%)	40 (100%)	3 (7.5%)
Ram 2021 [16]	60	61.0 ± 13.0	30 (50.0%)	24 (40.0%)	20 (33.3%)	44 (73.3%)	NA	NA
Minakata 2004 [17]	56	42.0 ± 20.0	23 (41.1%)	NA	11 (19.6%)	46 (82.1%)	37 (66%)	19 (33.9%)
Raffa 2022 [18]	66	58.4 ± 12.5	29 (43.9%)	NA	37 (40.9%)	51 (77.3%)	66 (100%)	22 (33.3%)
Bogachev-Prokophiev 2019 [19]	40	49.6 ± 14.3	14 (35.0%)	NA	NA	27 (67.5%)	40 (100%)	NA
Dorobantu 2022 [20]	83	52.0 ± 14.0	51 (61.4%)	NA	26 (31.3%)	49 (59.0%)	83 (100%)	NA
Ferrazzi 2015 [21]	39	58.0 ± 13.0	NA	NA	13 (33.3%)	32 (82.1%)	39 (100%)	NA
Schoendube 1995 [22]	58	48.2 ± 12.6	38 (65.5%)	NA	2 (3.4%)	53 (91.3%)	58 (100%)	NA
Zyrianov 2023 [23]	226	53.1 ± 14.2	127 (56.2%)	NA	44 (19.5%)	178 (78.8%)	NA	NA
Total	692	53.0 ± 14.8	341/653 (52.2%)	39/100 (39.0%)	158/628 (25.2%)	541/692 (78.2%)	387/406 (95.3%)	44/162 (27.2%)

N, number; NA, not available; NYHA, New York Heart Association.

### 3.2 Operative Technique

All patients underwent intraoperative transesophageal echocardiography to evaluate mitral valve structure, function, and the amount of myocardium to be resected. A median sternotomy was performed. Standard cardiopulmonary bypass was established through ascending aortic and right atrial cannulation. For some patients requiring secondary surgery, cardiopulmonary bypass was established through femoral artery cannulation. Myocardial protection was achieved by an intermittent antegrade or retrograde infusion of cardioplegia. All patients underwent aortotomy as an approach, and the aorta was transected about 10 mm above the right coronary artery ostium to allow observation of the left ventricular outflow tract. Septectomy was performed at the nadir of the right cusp, about 5 mm below the aortic valve, to the left of the trigon, and the thickness of the resected wedge-shaped interventricular septum was 1/3 to 1/2 of the base thickness. The excision was extended to the point of insertion of the papillary muscle with minimally invasive instruments [24].

Submitral valve repair mainly includes the following methods: false chordae and/or secondary chordae amputation, papillary muscle release or accessory papillary muscle resection, trabeculectomy between the septum and mitral valve apparatus, and separation of hypertrophic papillary muscles. The attachment to the leading edge of the anterior mitral leaflet was preserved to avoid iatrogenic mitral valve injury. After cessation of cardiopulmonary bypass, a provocation test was performed, and the left ventricular outflow tract gradient, systolic anterior motion, and mitral regurgitation were evaluated by intraoperative transesophageal ultrasound. If a residual gradient ≥30 mmHg, grade III and above mitral regurgitation, ventricular septal perforation, left ventricular wall rupture, or aortic valve perforation was found, cardiopulmonary bypass was performed to continue the operation.

### 3.3 Echocardiographic Analyses and Clinical Outcomes

During the operation, secondary aortic clipping was performed three times, including the repair of a left ventricular free wall rupture (n = 1) and residual left ventricular outflow track obstruction (LVOTO) (n = 2). Concomitant surgery for the previous etiology included resection of subaortic stenosis (n = 2), aortic valve repair (n = 6), aortic valve replacement (n = 3), radical pericardiectomy (n = 1), repair of Ebstein anomaly (n = 1), coronary artery bypass grafting (CABG) (n = 9), tricuspid valve repair (n = 2), and atrial septal defect closure (n = 2). No patient required mitral valve replacement. There were five (0.7%) deaths in the perioperative period, and the causes of death were infectious multiple organ failure (n = 1), failed septal myectomy combined with coronary artery bypass grafting (n = 1), gastrointestinal bleeding with cardiogenic shock (n = 1), refractory sepsis (n = 1), and left ventricular diastolic failure (n = 1).

The average cardiopulmonary bypass time was 44 ± 14.8 minutes, and the aortic clamping time was 64.7 ± 22.2 minutes. The average follow-up time was 39.6 ± 36.3 months, and the completion rate was 98.6%. Compared with baseline levels, left ventricular outflow tract gradient (83.6 ± 32.2 mmHg vs. 11.0 ± 7.8 mmHg, *p* < 0.01), maximum interventricular septal thickness (22.5 ± 5.1 mm vs. 14.7 ± 5.5 mm, *p* < 0.01), III/IV mitral regurgitation (351/692 vs. 17/675, *p* < 0.01), and systolic anterior motion (SAM) (181/194 vs. 11/215, *p* < 0.01) were significantly improved. The anterior mitral leaflet (AML)-annulus ratio was (0.49 ± 0.14 vs. 0.60 ± 0.12, *p* < 0.01), and the tenting area was (2.72 ± 0.60 ${\mathrm{cm}}^{2}$ vs. 1.95 ± 0.60 ${\mathrm{cm}}^{2}$, *p* < 0.01), suggesting that the mitral valve junction is far away from the left ventricular outflow tract. 26 (6.1%) patients required permanent pacemaker implantation for a complete atrioventricular block. 7 patients received ICD implantation due to a 24-hour ECG showing non-sustained tachycardia (n = 4) and a family history of sudden death (n = 3). 14 (2.1%) patients were in New York Heart Association functional class III/IV, significantly improved compared with preoperative (541/692 vs. 14/682, *p* < 0.01). 8 patients had mild aortic regurgitation (Tables 2,3, Ref. [10, 15, 16, 17, 18, 19, 20, 21, 22, 23]).

**Table 2.** **Preoperative versus follow-up echocardiographic analysis of patients**.

Author		LVOT gradient (mmHg)	Ventricular septal thickness (mm)	III/IV mitral regurgitation	Systolic anterior motion	LV ejection fraction (%)	AML-annulus ratio
Afanasyev [10]							
	Preoperative	86.4 ± 26.1	26.0 ± 1.5	24 (100%)	24 (100%)	71.8 ± 8.1	NA
	Follow-up	11.1 ± 4.9	18.1 ± 1.8	0	2 (8.3%)	63.2 ± 9.2	NA
Liu [15]							
	Preoperative	96.7 ± 23.3	17.0 ± 3.1	40 (100%)	40 (100%)	66.3 ± 3.9	NA
	Follow-up	8.8 ± 5.0	13.5 ± 1.8	0	0	NA	NA
Ram [16]							
	Preoperative	91.0 ± 39.0	24.8 ± 6.3	30 (50.0%)	NA	65.1 ± 2.0	0.75 ± 0.12
	Follow-up	13.0 ± 8.0	13.0 ± 2.9	1 (1.7%)	NA	65.0 ± 2.2	0.79 ± 0.11
Minakata [17]							
	Preoperative	97.0 ± 34.0	NA	36 (64.3%)	NA	71.0 ± 5.7	NA
	Follow-up	11.0 ± 11.0	NA	5 (9.3%)	NA	72.0 ± 6.7	NA
Raffa [18]							
	Preoperative	89.7 ± 34.5	18.9 ± 3.7	37 (56.1%)	53 (80.3%)	64.2 ± 7.1	NA
	Follow-up	15.4 ± 8.5	14.0 ± 2.6	1 (1.5%)	2 (3.1%)	NA	NA
Prokophiev [19]							
	Preoperative	92.3 ± 16.9	26.8 ± 4.5	40 (100%)	40 (100%)	76.2 ± 7.5	NA
	Follow-up	9.1 ± 2.4	15.4 ± 2.3	4 (10.0%)	2 (5.0%)	66.2 ± 7.4	NA
Dorobantu [20]							
	Preoperative	93.0 ± 33.0	24.0 ± 6.0	32 (38.6%)	NA	63.0 ± 5.0	NA
	Follow-up	13.0 ± 11.0	13.0 ± 11.0	1 (1.2%)	NA	59.0 ± 5.0	NA
Ferrazzi [21]							
	Preoperative	82.0 ± 43.0	17.0 ± 1.0	9 (23.1%)	NA	68.0 ± 6.0	0.45 ± 0.08
	Follow-up	9.0 ± 5.0	14.0 ± 2.0	1 (2.5%)	NA	63.0 ± 5.0	0.57 ± 0.08
Schoendube [22]							
	Preoperative	79.0 ± 33.0	25.0 ± 5.0	38 (65.5%)	24 (100%)	NA	NA
	Follow-up	5.0 ± 7.0	13.0 ± 4.0	0	5/49 (10.2%)	NA	NA
Zyrianov [23]							
	Preoperative	70.3 ± 25.2	23.0 ± 3.4	65 (28.8%)	NA	65.7 ± 6.0	0.43 ± 0.03
	Follow-up	11.0 ± 5.7	16.1 ± 3.6	4 (1.8%)	NA	63.0 ± 6.0	0.55 ± 0.06
Total							
	Preoperative	83.6 ± 32.2	22.5 ± 5.1 (n = 602)	351/692 (50.7%)	181/194 (93.3%)	66.7 ± 6.7 (n = 634)	0.49 ± 0.14 (n = 311)
	Follow-up	11.0 ± 7.8	14.7 ± 5.5 (n = 621)	17/675 (2.5%)	11/215 (5.1%)	63.4 ± 7.1 (n = 524)	0.60 ± 0.12 (n = 310)
		*p * < 0.01	*p * < 0.01	*p * < 0.01	*p * < 0.01	*p * < 0.01	*p * < 0.01

NA, not available; LVOT, left ventricular outflow tract; LV, left ventricle; AML, anterior mitral leaflet.

**Table 3.** **Follow-up clinical outcomes of patients**.

Author	Perioperative mortality	NYHA functional class III/IV	Pacemaker implantation	Hospital stay (days)	Unplanned reoperation	Atrial fibrillation
Afanasyev [10]	0	0	0	NA	0	NA
Liu [15]	0	0	2 (5.0%)	5.9 ± 0.2	2 (5.0%)	2 (5.4%)
Ram [16]	0	5 (8.3%)	5 (8.3%)	6.0 ± 0.5	0	20 (33.3%)
Minakata [17]	0	0	3 (5.4%)	12 ± 10	0	11 (20.4%)
Raffa [18]	1 (1.5%)	3 (4.6%)	3 (4.6%)	10.6 ± 8.3	1 (1.5%)	33 (50.8%)
Prokophiev [19]	0	0	2 (5.0%)	NA	1 (2.5%)	NA
Dorobantu [20]	1 (1.2%)	0	8 (9.8%)	NA	0	13 (15.8%)
Ferrazzi [21]	0	0	NA	NA	0	3 (7.7%)
Schoendube [22]	2 (3.4%)	4 (7.1%)	3 (5.4%)	NA	0	3 (5.4%)
Zyrianov [23]	1 (0.4%)	2 (0.9%)	NA	NA	0	15 (6.7%)
Total	5 (0.7%)	14/682 (2.1%)	26/427 (6.1%)	8.7 ± 7.2 (n = 222)	4/687 (0.6%)	100/618 (16.2%)

NA, not available; NYHA, New York Heart Association.

During the follow-up period, 10 patients died, and the causes of death included: chronic respiratory failure (n = 1), congestive heart failure (n = 6), and renal failure (n = 3). There were 4 unplanned reoperations: one patient was readmitted for mitral annuloplasty and posterior leaflet plication due to residual left ventricular outflow tract gradient and mitral regurgitation (n = 1); endocarditis (n = 1); repair of aortic perforation (n = 1); and repair of ventricular septal perforation (n = 1).

## 4. Discussion

The postoperative and follow-up data from ten studies were pooled, and it was found that: (1) Ventricular septal myectomy combined with sub-mitral valve repair significantly reduces the pressure gradient of the left ventricular septal outflow tract, eliminates the SAM phenomenon, improves mitral regurgitation, and relieves heart failure in patients with HOCM with severe left ventricular outflow tract obstruction and mitral valve regurgitation. (2) Patients do not require additional mitral valve intervention, and only 0.6% of patients require reoperation for secondary mitral valve surgery after the procedure. (3) Retaining a certain thickness of the ventricular septum during the operation can also effectively eliminate the obstruction and avoid surgical adverse events such as ventricular septal perforation and ventricular septal rupture. (4) However, after ventricular septal resection combined with subvalvular management, the proportion of patients requiring permanent pacemaker implantation is high.

The classic Morrow operation involves making two parallel incisions in the interventricular septum. However, due to the limited field of view and operating range of surgical exposure, some patients with non-outflow tract hypertrophy, such as apical hypertrophy, cannot achieve the expected results. Later, an extended myectomy was proposed, which involves extending the range of the surgery to both sides and the apex. Currently, modified Morrow surgery is the preferred surgical strategy for patients with HOCM [24]. Sufficient ventricular septal resection is effective for most patients, but there are some limitations, especially for patients with a thin ventricular septum and subvalvular structural abnormalities. Mitral valve replacement has also been proposed as an alternative treatment for HOCM, but due to the durability of artificial valves and the high incidence of infection, thromboembolism, and other problems, it is used less frequently at present [25].

Hypertrophy of the papillary muscles, shortening and thickening of the secondary chordae, and fibrosis in patients with hypertrophic cardiomyopathy may lead to abnormal tethering of the anterior mitral leaflet and poor coaptation of the anterior and posterior mitral leaflets. During systole, mitral commissures move toward the left ventricular outflow tract, increasing SAM-mediated mitral regurgitation [23, 26, 27, 28]. In particular, for patients with insignificant ventricular septal hypertrophy but with left ventricular outflow tract obstruction, ventricular septal hypertrophy may not be the primary cause of the obstruction [29]. Moreover, the SAM phenomenon cannot be fully explained by the Venturi effect [30]. The contribution of the mitral valve device and all its components to the dynamic obstruction of the LVOT varies; thus, surgical correction is recommended in addition to extended myectomy for optimal results [31, 32]. After subvalvular repair, the anterior mitral leaflet-annulus ratio increases, and the tenting area decreases. This helps the anterior leaflet of the mitral valve move backwards and promotes the coaptation plane of the anterior and posterior leaflets to move backwards away from the left ventricular outflow tract, thereby eliminating the SAM phenomenon, relieving mitral valve regurgitation, preventing left ventricular outflow tract obstruction, and avoiding mitral valve replacement [21].

The study conducted by Liu *et al*. [15] compared the one-year follow-up results of the combined group (n = 40) and the isolated septal myectomy group (n = 106). The study found that when there was no significant difference in postoperative ventricular septal thickness, the combined group could better improve the SAM and mitral regurgitation (MR) levels, and the left ventricular outflow tract gradient was lower. These results are consistent with the results of [19, 21]. There was no significant difference in aortic clipping time between the combined group and the isolated septal myectomy group (38.0 ± 7.1 minutes vs. 35.6 ± 7.3 minutes, *p* = 0.076). In addition, the risk of secondary aortic cross-clamping in the isolated septal myectomy group was significantly higher (odds ratio [OR] 1.24, 95% confidence interval [CI] 1.02–14.51, *p* = 0.02) [15, 16, 19]. This risk may also be related to the surgical experience.

Aortic regurgitation is a common complication after septal resection, and the incidence in this study was found to be only 1.2% (n = 8). The incidence of mitral valve replacement was even lower, at only 0.3% (n = 2). Despite a wider scope of surgical intervention, the incidence of ventricular septal perforation and defect was also low, at 0.3% (n = 2). During the follow-up period, the incidence of atrial fibrillation was 16.2%. These clinical outcomes were comparable to those seen after isolated septal myectomy [33, 34, 35]. For patients with mild septal hypertrophy but severe SAM and MR, subvalvular management is a better option than mitral valve replacement. Combined surgery can effectively eliminate LVOTO and alleviate MR, reducing the risk of iatrogenic ventricular septal perforation or defect. Additionally, it can lower the incidence of intraoperative repeat aortic clipping [19, 29, 36]. However, due to the wider range of myocardium involved in combined surgery, it can have a greater impact on the normal rhythm conduction of the ventricle. The rate of permanent pacemaker implantation during hospitalization for surgical patients was 6.1%, which is higher than the rate of 3.5% seen in patients with a simple septal myectomy. Excluding the patients with preoperative right bundle branch block, only 1.1% of patients with normal preoperative ECG evaluation required permanent pacemaker implantation [37]. Therefore, surgeons need to screen for right bundle branch block before combined surgery to avoid sudden complete atrioventricular block after surgery.

According to the Mayo Clinic’s experience [38], only 2.1% of patients without intrinsic mitral valve disease required additional mitral valve intervention. In a comparison of Doppler echocardiographic findings before and after 1830 isolated diaphragm resections without congenital mitral valve disease, the number of class III/IV patients decreased from 54.3% to 1.7%. In 2019, the American Society of Thoracic Surgeons analyzed septal myectomy data from over 2300 patients, of whom approximately one-third (n = 801) underwent septal myectomy combined with mitral valve intervention. Mitral valve repair was performed in 62% of cases, while mitral valve replacement was performed in the remaining 38% [39]. For mitral valve intervention, mitral valvuloplasty is prioritized over mitral valve replacement, and the 2-year survival rate of mitral valve repair is much better than that of mitral valve replacement (96.7% vs. 87.2%, *p* < 0.05) [40]. According to a systematic review, mitral valve repair has several advantages over mitral valve replacement, including a lower risk of death, dysfunction of the mitral valve after the operation, reoperation on the mitral valve, and thromboembolic events [41].

Zyrianov *et al*. [23] retrospectively analyzed 212 patients with HOCM who underwent septal myectomy combined with secondary chordal cutting. Based on the thickness of the ventricular septum, the patients were divided into two groups: the mild ventricular septal hypertrophy group (<20 mm, n = 62) and the severe ventricular septal hypertrophy group (>20 mm, n = 150). The echocardiographic evaluations of the two patient groups were compared to those of 124 normal individuals. The degree of mitral valve displacement to the left ventricular outflow tract was similar in both groups before the operation. This suggests that septal thickness is not the main factor influencing SAM, but that secondary chordae are also involved, with abnormal secondary chordae pulling the anterior mitral leaflet toward the left ventricular outflow tract. There were no significant differences in postoperative clinical characteristics between the two groups, except for the preserved septal thickness (17 ± 4 mm vs. 14 ±2 mm, *p* < 0.01). The increase in the degree of the AML annulus ratio after the operation was similar in both groups (+0.11 ± 0.06 vs. +0.12 ± 0.07, *p* = 0.780). The reduction in the degree of mitral valve tenting area was also similar in both groups (–0.73 ± 0.61 vs. –0.81 ± 0.52, *p* = 0.150). The proportion of AML-annulus ratio (52% vs. 45%, *p* = 0.150) and tenting area (54% vs. 52%, *p* = 0.711) returning to the normal range after surgery was similar in both groups. In contrast, in a study by Ferrazzi *et al*. [21], patients with mild ventricular septal hypertrophy (IVS <19 mm) did not have a significant change in the relative mitral valve position after isolated myectomy. This indicates that the combined procedure is effective in eliminating SAM and improving mitral regurgitation in patients with hypertrophic cardiomyopathy (HCM), regardless of the degree of preoperative septal hypertrophy [18, 23]. This result may be due to the release of the secondary chordae to the anterior leaflet of the mitral valve and the originally loose primary chordae being tightened during systole to prevent the mitral valve leaflets from approaching the interventricular septum. HOCM is often accompanied by prolongation of the anterior mitral valve leaflet, but the length of the increased anterior mitral valve leaflet has nothing to do with the gradient of the left ventricular outflow tract. These observations suggest that the length of the anterior mitral valve leaflet is not a major factor affecting the surgical strategy [42].

In a randomized controlled study, 48 patients were assigned to undergo either ventricular septal myectomy with edge-to-edge repair or secondary chordal cutting [10]. Postoperative Doppler echocardiography revealed no significant difference in the left ventricular outflow tract gradient (15.4 ± 7.6 mmHg vs. 11.1 ± 4.9 mmHg, *p* = 0.078) between the cutting group and the edge-to-edge (E2E) group. However, the peak transmitral pressure gradient (TPG) (4.7 ± 2.8 mmHg vs. 7.8 mmHg, *p* = 0.014) and the average TPG (2.1 ± 2.8 mmHg vs. 3.9 ± 1.7 mmHg, *p* = 0.013) were lower in the cutting group compared to the E2E group. The proportion of patients with mild residual mitral regurgitation was higher in the cutting group (25% vs. 0%). The E2E technique was associated with mild postoperative mitral stenosis, whereas secondary chordal cutting was associated with postoperative mild residual mitral regurgitation. Therefore, the institution chose to use secondary chordal resection as the preferred surgical method because it could avoid an additional left atrial incision and reduce the risk of postoperative mitral stenosis. However, the durability of the mitral valve after E2E versus a normal mitral valve is not yet clear, especially in younger patients. Inappropriate stitch placement during the E2E procedure not only makes it difficult to correct MR and LVOTO caused by anterior mitral leaflet anterior displacement but also leads to secondary aortic clipping [43]. Septal myectomy combined with mitral valve extension utilizes an autologous pericardium or bovine pericardial patch to expand and reinforce the mitral valve, which also achieves the goal of eliminating left ventricular outflow tract obstruction and improving mitral regurgitation. Vriesendorp *et al*. [9] reported the 15-year follow-up results of 98 postoperative patient. The 1-, 5-, 10-, and 15-year cumulative survival rates were 98%, 92%, 86%, and 83%, respectively. Patients with non-obstructive hypertrophic cardiomyopathy had cumulative survival rates of 98%, 97%, 88%, and 83% (*p* = 0.8). Age- and gender-matched normal individuals had cumulative survival rates of 99%, 97%, 92%, and 85% (*p* = 0.3). There was no significant difference between the three groups. However, this technique also faces the problem of recurrent mitral regurgitation caused by the degradation or splitting of the pericardial patch. The resection-plication-release (RPR) technique has also been proposed for the treatment of patients with HOCM with elongated anterior mitral leaflets [44].

The secondary chordae play an important role in maintaining the geometry of the left ventricle, and their resection may affect ventricular contraction [45, 46]. From pooled data, although the left ventricular ejection fraction is reduced (66.7 ± 6.7 vs. 63.4 ± 7.1, *p* < 0.01), it does not affect left ventricular function. In Zyrianov’s study [23], patients in the severe group had a higher septal resection thickness than those in the mild group (32% vs. 22%). The mild group had no significant changes in left ventricular end-diastolic volume (88 ± 29 mL vs. 89 ± 23 mL, *p* = 0.86) but an increased left ventricular end-systolic volume (28 ± 11 mL vs. 33 ± 12 mL, *p* < 0.01) after septal myectomy combined with secondary chordal cutting. In the severe group, both the end-diastolic volume (95 ± 30 mL vs. 102 ± 29 mL, *p* = 0.01) and end-systolic volume (33 ± 14 mL vs. 38 ± 15 mL, *p* < 0.01) increased [23]. This shows that the volume of the left ventricle increased after the operation, but the systolic function weakened. However, the improvement of symptoms in the mild ventricular septal hypertrophy group does not depend on the volume of the left ventricle, which may be related to the relief of left ventricular outflow tract obstruction.

Isolated septal resection generally requires a resection of 40%–50% of the maximum septal thickness [47], and even in some institutions, only 10 mm of the septum is preserved [48]. This often represents a great surgical difficulty and increases the risk of septal perforation or even rupture in patients. In contrast, combined surgical resection of 30% of the ventricular septum was considered sufficient in Zyrianov’s study [23], even in patients with moderate to severe hypertrophy (IVS >20 mm). This further demonstrates the benefit of sub-mitral valve repair [36]. For patients with HCM and severe MR, it is necessary to identify the structure of the mitral valve apparatus by cardiac magnetic resonance (CMR) or trans esophageal echocardiography (TEE). If the mitral valve and subvalvular structure are abnormal or the thickness of the ventricular septum is thin, it is recommended to perform superficial septal muscle myectomy combined with sub-mitral valve repair, which can effectively eliminate the gradient of the left ventricular outflow tract, improve mitral regurgitation, and have a low incidence of adverse events.

The limitation of this study is that it included 8 retrospective analyses and 2 randomized controlled trials. Most of them were retrospective studies, and there was no control group. Moreover, the sample size was limited, and the inclusion criteria were not absolutely uniform, leading to certain selection bias, attrition bias, and missing outcome variable bias. Individual patients had concomitant surgery, which also had a certain impact on the statistics of the outcome. Randomized controlled trials represent a higher level of evidence, but they are less feasible and less ethical for surgical studies. In this case, non-randomized and observational studies are also valuable evidence.

Sub-mitral valve repair surgery has greater flexibility, and surgeons mostly operate according to personal experience and preference. When experienced surgeons perform ventricular septal myectomy, the mortality rate is less than 1%, and the clinical success rate is 90%–95%. Maron summarized five major North American clinics from 2000 to 2014, including the Mayo Clinic and Cleveland Clinic [49]. Among the 3695 patients in high-volume hypertrophic cardiomyopathy surgery centers, the mortality rate was only 0.4%, while the mortality rate of patients in low-volume HCM surgery centers in the United States was about 5.9% (n = 665) during the same period [50]. Surgical outcomes and adverse event rates in cardiac surgery centers with different volumes are quite different. Accurately judging whether septal resection combined with sub-mitral valve repair has significant advantages compared with other surgical methods requires a larger sample size and longer follow-up.

## 5. Conclusions

The mechanism of left ventricular outflow tract obstruction in hypertrophic cardiomyopathy is very complex. It involves different factors such as the segmental hypertrophic interventricular septum, hypertrophic and displaced papillary muscles, fibrotic and shortened chordae, thickened and elongated mitral valve leaflets, and even deranged myocardial trabeculae. A septal myectomy combined with sub-mitral management represents a comprehensive surgical approach to correct left ventricular outflow tract obstruction and mitral regurgitation. This approach targets pathological mechanisms such as septal hypertrophy and sub-mitral structural abnormalities, resulting in good surgical results and long-term survival.

## Data Availability

All data and materials were from published researches.

## Abbreviations

SAM, systolic anterior motion; LVOT, left ventricular outflow tract; MR, mitral regurgitation; IVS, interventricular septum; AML, anterior mitral leaflet; LVOTO, left ventricular outflow track obstruction; ICD, implantable cardioverter defibrillator; E2E, edge to edge; TPG, transmitral pressure gradient; RPR, resection-plication-release; RCT, Randomized Controlled Trial; CABG, coronary artery bypass grafting; CMR, cardiac magnetic resonance; TEE, trans esophageal echocardiography.

## Supplementary Material

Supplementary material associated with this article can be found, in the online version, at https://doi.org/10.31083/j.rcm2409268.
