# Supplementary material for: Septal Myectomy and Subvalvular Repair in Hypertrophic Cardiomyopathy, a Systematic Review and Pooled Analysis
Source: Rev Cardiovasc Med. 2023 Sep 22;24(9):268. doi: 10.31083/j.rcm2409268 (PMC11262436; doi:10.31083/j.rcm2409268)
Supplement: Supplementary file 1 [file 2153-8174-24-9-268-s1.zip › 2153-8174-24-9-268-s1.docx]

Supplementary Material, Table S1. MINORS assessment of included studies

| **Methodological items for non-randomized studies** | Liu | Ferrazzi | Prokophiev | Ram | Afanasyev | Schoendube | Minakata | Dorobantu | Raffa | Zyrianov |
| --- | --- | --- | --- | --- | --- | --- | --- | --- | --- | --- |
| 1. A clearly stated aim | 2 | 2 | 2 | 2 | 2 | 2 | 2 | 2 | 2 | 2 |
| 2. Inclusion of consecutive patients | 2 | 1 | 2 | 1 | 2 | 1 | 1 | 2 | 1 | 1 |
| 3. Prospective collection of data | 0 | 0 | 2 | 0 | 2 | 0 | 0 | 0 | 0 | 0 |
| 4. Endpoints appropriate to the aim of the study | 1 | 1 | 1 | 1 | 1 | 1 | 1 | 1 | 1 | 1 |
| 5. Unbiased assessment of the study endpoint | 2 | 2 | 2 | 2 | 2 | 2 | 2 | 2 | 2 | 2 |
| 6. Follow-up period appropriate to the aim of the study | 2 | 2 | 2 | 2 | 2 | 2 | 2 | 2 | 2 | 2 |
| 7. Loss to follow up less than 5% | 2 | 2 | 2 | 2 | 2 | 2 | 2 | 2 | 2 | 2 |
| 8. Prospective calculation of the study size | 0 | 0 | 0 | 0 | 0 | 0 | 0 | 0 | 0 | 0 |
| **Additional criteria in the case of comparative study** |  |  |  |  |  |  |  |  |  |  |
| 9. An adequate control group | 2 | 2 | 2 | 2 | 2 |  |  |  |  |  |
| 10. Contemporary groups | 0 | 0 | 0 | 0 | 0 |  |  |  |  |  |
| 11. Baseline equivalence of groups | 2 | 2 | 2 | 2 | 2 |  |  |  |  |  |
| 12. Adequate statistical analyses: | 2 | 1 | 2 | 2 | 2 |  |  |  |  |  |
| Total | 17 | 16 | 19 | 16 | 19 | 10 | 10 | 11 | 10 | 10 |

MINORS, methodological index for non-randomized studies;
